# Basal ganglia: physiological, behavioral, and computational studies

**DOI:** 10.3389/fnsys.2014.00150

**Published:** 2014-08-21

**Authors:** Ahmed A. Moustafa, Izhar Bar-Gad, Alon Korngreen, Hagai Bergman

**Affiliations:** ^1^Department of Veterans Affairs, New Jersey Health Care System, School of Social Sciences and Psychology, Marcs Institute for Brain and Behaviour, University of Western SydneySydney, NSW, Australia; ^2^Gonda Brain Research Center, Bar-Ilan UniversityRamat Gan, Israel; ^3^Everard Goodman Faculty of life sciences, Bar-Ilan UniversityRamat Gan, Israel; ^4^Department of Neurobiology (Physiology), Faculty of Medicine, Edemond and Lily Safra Center for Brain Research, Institue of Medical Research Israel-Canada, The Hebrew University of JerusalemJerusalem, Israel

**Keywords:** basal ganglia, dopamine, Parkinson's disease (PD), computational modeling, animal studies, human imaging studies, deep brain stimulation

The basal ganglia has received much attention over the last two decades, as it has been implicated in many neurological and psychiatric disorders, including Parkinson's disease (PD), Attention Deficit Hyperactivity Disorder (ADHD), Tourette's syndrome, and dystonia. Most of current basal ganglia research—in both animals and humans—attempts to understand the neural and biochemical substrates of basic motor and learning processes, and how these are affected in human patients as well as animal models of brain disorders, particularly PD.

The current volume contains research articles and reviews describing basic, pre-clinical and clinical neuroscience research of the basal ganglia written by researchers of the basal ganglia and attendees of the 11th Triennial Meeting of the International Basal Ganglia Society (IBAGS) that was held on March 3–7th, 2013 at the Princess Hotel, Eilat, Israel. Specifically, articles in this volume include research reports on the biochemistry, computational theory, anatomy, and physiology of single neurons and functional circuitry of the basal ganglia networks.

Below, we provide a summary of articles published in the volume. We divided the articles into 4 sections: animal studies, human studies, computational modeling, and reviews.

## Animal studies

Using physiological recordings, Adler et al. (2013) studied the relationship of both medium spiny cells and interneurons of the striatum while monkeys were performing a Pavlovian conditioning task, showing that both classes of neurons play a key role in conditioning. Along these lines, Yael et al. (2013) studied the effects of D2 antagonists (haloperidol) on striatal activity, finding evidence that it alters firing patterns of medium spiny cells and interneurons. Interestingly, Bronfeld et al. (2013) found that microinjection of the GABA_A_ antagonist bicuculline in the dorsal striatum leads to Tourette's tics in rats, potentially highlighting a role of interneurons in such motor symptoms. However, future studies should explain the exact function of interneurons in the generation of tics. In another study, Plata et al. (2013) found that the administration of nicotine activates inhibitory interneurons in the striatum, which can treat motor symptoms of PD (as similar to dopaminergic medications). The implications of these findings remain to be shown in clinical studies. Using whole-cell patch-clamp recordings, Arias-Garcia et al. (2013) found, for the first time, that Ca^2+^-activated K^+^ channels explain the different durations between corticostriatal responses in the basal ganglia direct and indirect pathways. Future research should explain the relationship between these findings and motor symptoms in PD.

While the above studies focus on the striatum, other studies in the volume address the function of other downstream basal ganglia structures. For example, Lavian et al. (2013) studied the effects of high and low frequency stimulation of the subthalamic nucleus on the activations of subthalamic and globus pallidus neurons *in vitro*, finding evidence of complex relationship between these two basal ganglia structures. In another physiological study, Espinosa-Parrilla et al. (2013) recorded from the subthalamic nucleus in behaving monkeys, showing evidence that it plays a role in reward and motivational processes; their findings suggest that the subthalamic nucleus transforms motivational information into motor responses. These findings can potentially shed light on how motivational factors impact motor symptoms in PD. Benhamou and Cohen (2014) studied the types and activity patterns of globus pallidus internal segment neurons, finding evidence of the existence of distinct neuronal populations in this brain structure. Future physiological studies should investigate the exact function of these cell populations, and whether they are differentially impacted by neurological disorders.

Other studies in the volume focused on behavioral profile in animal models of PD. In one study, Pasquereau and Turner (2013) investigated the effects of fast muscle stretches on the primary motor cortex activity in monkeys before and after MPTP treatment; they found, for the first time, evidence that MPTP can alter primary motor cortex response to muscle stretch. Future research should investigate if these findings can shed light on the nature of akinesia and bradykinesia symptoms in patients with PD. In another study, Grieb et al. (2013) video monitored locomotor behavior in 6-OHDA rats, finding evidence of impaired motion speed among other locomotion variables. These data replicate findings from human studies with PD.

## Human studies

Most of the studies in the volume focus on PD. For instance, Moshel et al. (2013) have investigated the pattern of subthalamic nucleus oscillation and synchronicity in PD patients during the deep brain stimulation procedure. This is among the few studies that investigate the physiological patterns of different regions within the subthalamic nucleus in PD patients. Along the same lines, Eitan et al. (2013) recorded from the subthalamic nucleus in PD patients during deep brain stimulation procedures while subjects were presented with emotional stimuli and found evidence for hemispheric (right) and domain (bentro-medial) specificity of these responses. Future research should investigate hemispheric specificity of cognitive and motor processes in PD patients undergoing deep brain stimulation.

Other studies in the volume focus on cognitive profiles of PD patients. Filoteo and Maddox (2014) studied category learning performance in PD patients, finding evidence for category discontinuity on learning. These extends prior findings by the same authors on the effects of PD on category learning (Filoteo et al., 2007). Using imaging and computational modeling, O'Callaghan et al. (2013) studied learning impairment in PD patients, showing evidence for a role for the ventromedial prefrontal cortex and inferior frontal gyrus in these processes; this extends prior findings of the role of the basal ganglia in learning processes (Bodi et al., 2009; Keri et al., 2010).

In one study, Moll et al. (2014) recorded single cell as well as local field potential activity from globus pallidus (internal and external segments) in patients with cervical dystonia undergoing deep brain stimulation. They found that cervical dystonia is associated with asymmetric pallidal functions. However, future experimental and theoretical work should explain how damage to the globus pallidus relates to dystonia symptoms, and whether the basal ganglia direct pathway plays a similar role in dystonia symptoms.

## Computational modeling

The volume contains different kinds of computational models of the basal ganglia that focus on physiological properties of basal ganglia structures or the effects of PD on motor and cognitive processes.

Regarding physiological models, Brody and Korngreen (2013) provided a compartment computational model linking synaptic plasticity with globus pallidus dynamics, as well as understanding the mechanism of the differential effects of low and high frequency stimulation on this structure. Future computational studies should also investigate the dynamics of other basal ganglia structures including low and high frequency stimulation of the subthalamic nucleus. Along the same lines, Merrison-Hort and Borisyuk (2013) developed a computational model of the globus pallidus along with afferent inputs from the cortex and subthalamic nucleus, highlighting how motor symptoms in PD can arise from aberrations to this circuit. This work shows that complex interactions among cortical and subcortical structures underlie the occurrence of motor symptoms in PD. Unlike the above models, Guo et al. (2013) provided a computational model of the thalamocortical circuit to investigate activity patterns of the thalamus in dystonia and PD, finding evidence that both diseases have to some extent similar effects on these basal ganglia structures. However, it remains to be shown on how damage to different basal ganglia structures can leads to different symptoms as in PD and dystonia.

Some other models in the volume focus on simulating basal ganglia-related motor and cognitive processes in health and disease. For example, Gupta et al. (2013) designed a basal ganglia model of precision grip in PD, addressing the effects of dopamine medications on grip function. This is among the first models that explains how dopamine medications impact grip force in PD patients. Future work should also address the effects of deep brain stimulation on grip force in PD. Muralidharan et al. (2014) provided one of the first computational models that simulate freezing of gait in PD as well as the effects of dopamine medications on gait parameters. This model specifically focused on simulating data from healthy subjects and PD patients passing through doorways of different widths. Future work should explain other factors that lead to freezing of gait, including obstacle avoidance, turning, and motor initiation. In another study, Balasubramani et al. (2014) designed a computational models of the role of dopamine and serotonin interaction in the basal ganglia in reward, punishment, and risk-based decision making, as studied previously in PD patients (Frank et al., 2007; Bodi et al., 2009). This is among the few models that investigated the function of dopamine and serotonin in behavioral processes in PD. As for motor processes, Tomkins et al. (2013) developed a model of the striatum in action selection and decision making, showing how this circuit decides on responses based on cortical inputs. Future work should investigate how action selection relates to motor symptoms in PD, including akinesia, bradykinesia, and medication-induced dyskinesia. Gershman et al. (2014) address the issue of time in reinforcement learning models of the basal ganglia, suggesting a single mechanism of reinforcement learning and interval timing. Paul and Ashby (2013) provided a computational model showing how memory systems (explicit and procedural memory systems) interact during learning.

## Reviews

Our volume includes various reviews, which focus on either the physiological properties of a basal ganglia structure, a basal ganglia-related disorder, computational models of the basal ganglia, mechanism of action of deep brain stimulation in basal ganglia-related disorders, as well as interaction of the basal ganglia with other brain structures.

For example, Schwab et al. (2013) provided a review of the physiological properties of the globus pallidus external segment in health and disease (focusing on PD). Along the same lines, Nambu and Tachibana (2014) also reviewed data on basal ganglia (particularly subthalamic nucleus, and globus pallidus) oscillations in relation to PD motor symptoms. These reviews complement many other existing review on the function and role of the striatum in PD motor symptoms. On the other hand, Molochnikov and Cohen (2014) reviewed data on the function of nigrostriatal and mesolimbic dopamine in different hemispheres. This review highlights the dissociable function of different hemispheres, yet future work should relate these findings to neurological and psychiatric disorders. In another review, Bosch-Bouju et al. (2013) reviewed data on the role of the thalamus in the integration of data from the cortex, cerebellum, and basal ganglia, and how such pathways play a role in the occurrence of motor symptoms in PD. In another paper, Nougaret et al. (2013) provided a commentary on a recent anatomical study on the prefrontal-subthalamic pathway in primates (Haynes and Haber, 2013). These studies highligh key findings on the patterns of connections from various prefrontal and cortical areas to the subthalamic nucleus. These studies have implications for understanding the motor and cognitive function of the subthalamic nucleus in healthy subjects as well as in patients with PD.

Further, some reviews in the volume attempt to explain the neural mechanism of deep brain stimulation. For instance, Chiken and Nambu (2014) reviewed studies arguing that deep brain stimulation work by disrupting abnormal signal transmission in PD, dystonia, and tremor, while Smith et al. (2014) reviewed data on the role of thalamo-striatal pathway in motor symptoms in PD, and suggest that deep brain stimulation to this pathway can aid in the treatment of PD symptoms. Unlike these reviews, Jahanshahi (2013) reviewed recent studies on the effects of subthalamic deep brain stimulation on motor and cognitive processes in PD, focusing on inhibitory and cognitive control. This review shows that beside motor processes, deep brain stimulation has a complex effect on cognitive control as well as other cognitive processes.

Some other reviews in the volume focus on behavioral processes. For instance, Seger (2013) provided an overview of the function of the visual cortico-striatal loop. This loop has so far received little attention in the literature than other basal ganglia loops. Interestingly, Simola et al. (2013) reviewed data on how early movement can impact abnormal involuntary movement and dyskinesia, focusing on the effects of dopamine replacement therapies on these motor complications. Studies reviewed here shed light on how levodopa and dopamine agonists can differentially affect the occurrence of dyskinesia in a subset of PD patients. Unlike prior reviews, Retailleau and Boraud (2014) reviewed data on the role of dopamine projection to the hippocampus in navigation in 6-OHDA rats. This review addresses an often less studies issues as most existing studies focus on dopamine projections to the basal ganglia and prefrontal cortex. In another review, Shine et al. (2013) provided a review of the neural and cognitive underpinnings of freezing of gait in PD. This review shed light on the complexity of freezing of gait, and explain how damage to the basal ganglia and the cortex can lead to lead to this motor symptom. Further, Moustafa and Poletti (2013) reviewed studies on the cognitive and neural abnormalities in subtypes of PD patients including tremor- vs. akinesia-dominant as well as patients with or without depression, impulsivity, and hallucinations. Further, Leisman et al. (2014) reviewed studies addressing the relationship between the basal ganglia and ADHD. This review explain how damage to corticostrial loops lead to attentional and impulsive behavior in ADHD patients.

Other reviews focus on computational models of the basal ganglia. In one study, Schroll and Hamker (2013) have provided an extensive review of existing basal ganglia models, focusing on models relating the anatomy of the basal ganglia to behavioral processes. This study reviewed most existing studies on the function of the basal ganglia direct and indirect pathways. On the other hand, Helie et al. (2013) reviewed basal ganglia network models of various motor and cognitive processes, including working memory, categorization, and sequence learning, and handwriting. This review shows how the basal ganglia can play a similar role in motor and cognitive processes.

## Conclusions

This volume provides the latest data on animal models of basal ganglia dysfunction and clinical studies in human patients with basal ganglia-related disorders. Although there are a multitude of studies on the anatomy, phyiology, and computational models of the basal ganglia, there are still many open questions. Future experimental and computational studies will continue to understand how exactly neurological and psychiatric disorders impact the basal ganglia as well as the neural mechanism of medications and deep brain stimulation.

### Conflict of interest statement

The authors declare that the research was conducted in the absence of any commercial or financial relationships that could be construed as a potential conflict of interest.
